# Transcriptome sequencing and gene expression analysis revealed early ovule abortion of *Paeonia ludlowii*

**DOI:** 10.1186/s12864-023-09171-1

**Published:** 2023-02-17

**Authors:** Ting-qiao Chen, Yue Sun, Tao Yuan

**Affiliations:** 1 Beijing Key Laboratory of Ornamental Plants Germplasm Innovation & Molecular Breeding, Beijing Laboratory of Urban and Rural Ecological Environment, Engineering Research Center of Landscape Environment of Ministry of Education, Key Laboratory of Genetics and Breeding in Forest Trees and Ornamental Plants of Ministry of Education, School of Landscape Architecture, National Engineering Research Center for Floriculture, Beijing Forestry University, Beijing, 100083 China; 2 School of Geography and Environmental Science/School of Karst Science, Guizhou Normal University, Guiyang, 550001 China

**Keywords:** *Paeonia ludlowii*, ovule abortion, transcriptome sequencing, difference analysis

## Abstract

**Background:**

*Paeonia ludlowii* (Stern & G. Taylor D.Y. Hong) belongs to the peony group of the genus *Paeonia* in the *Paeoniaceae* family and is now classified as a “critically endangered species” in China. Reproduction is important for this species, and its low fruiting rate has become a critical factor limiting both the expansion of its wild population and its domestic cultivation.

**Results:**

In this study, we investigated possible causes of the low fruiting rate and ovule abortion in *Paeonia ludlowii*. We clarified the characteristics of ovule abortion and the specific time of abortion in *Paeonia ludlowii*, and used transcriptome sequencing to investigate the mechanism of abortion of ovules in *Paeonia ludlowii*.

**Conclusions:**

In this paper, the ovule abortion characteristics of *Paeonia ludlowii* were systematically studied for the first time and provide a theoretical basis for the optimal breeding and future cultivation of *Paeonia ludlowii*.

**Supplementary Information:**

The online version contains supplementary material available at 10.1186/s12864-023-09171-1.

## Introduction

**Fig. 1:**
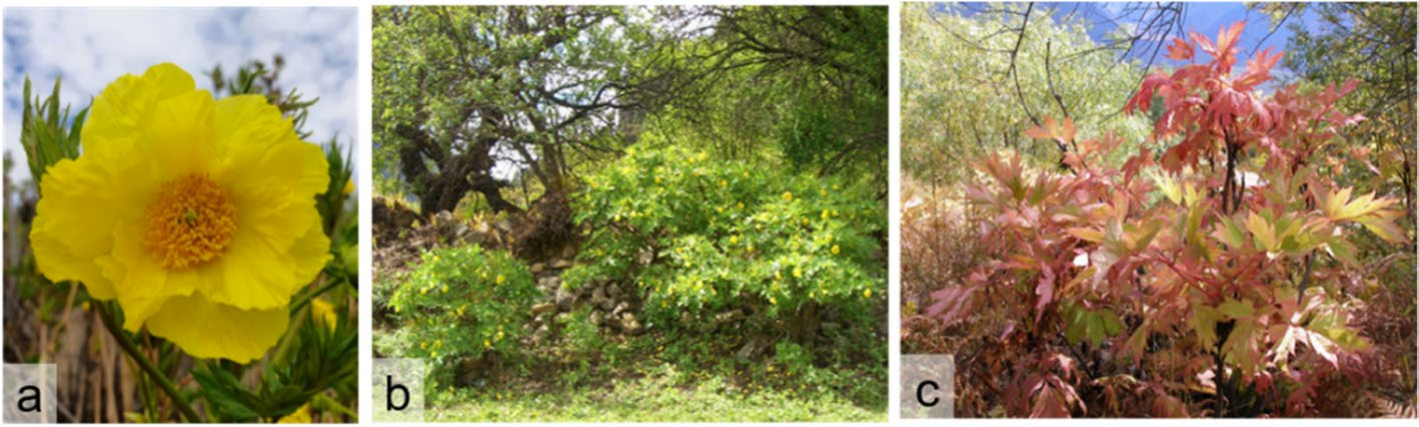
*Paeonia ludlowii* (Mirui Township, Bayi District, Nyingchi, Tibet); **a** and **b**: May 2021; **c**: October 2021

*Paeonia ludlo*wii (Stern & G. Taylor D. Y. Hong) is a wild endemic plant with conservation status II in China (National Forestry and Grassland Administration, 2021). It is a tall plant with multiple yellow flowers and it can develop secondary branches around florescence in spring which was distinguished from other species and cultivars of *Paeonia* Sect. It has breeding, medicinal, ornamental, and exploitative values [1–4] (Fig. 1). Wild *P.ludlowii* is distributed only in the Nyingchi region of Tibet, China [5], while *P.ludlowii* grown in other countries has been introduced from the Nyingchi region in China [6].

In the late twentieth and early twenty-first centuries, *P.ludlowii* was considered an independent species. Subsequently, its geographical distribution was further defined [5] and introduced in Gansu and Henan Provinces [7, 8]. Significant progress has been made to understand the genetic resources [9], diversity [10, 11], nutritional value and applications of *P.ludlowii* [3, 12–14], including endophytic fungi [15], seed germination, seedling establishment [16, 17], pollen germination, storage characteristics [18], and saline-alkali stress [19]. Its self and heterozygous affinity, but its fruit set rate is very low [7, 20, 21], resulting in a high seed defeat rate. However, no research has assessed seed defeat in *P.ludlowii*.

Other endangered plants such as dove trees (*Davidia involucrata*) [22], *Caryocar brasiliense* [23], and *Liriodendron chinense* [24] also experienced severe seed abortion. Recent studies have demonstrated that seed abortion is a complex plant behavior influenced by a combination of internal and external conditions [25]. As molecular biology theories and techniques have progressed, research related to seed abortion has gradually shifted from physiological and morphological levels to molecular and genetic levels. Currently, the main research focus is on the dove tree [22, 26], pomegranate (*Punica granatum*) [27], longan (*Dimocarpus longan* Lour.) [28], peanut (*Arachis hypogaea*) [29], hazelnut (*Corylus heterophylla*) [30, 31], grapes (*Vitis vinifera*) [32], rice (*Oryza sativa*) [33], and other species in which significant progress has been made identifying and obtaining some candidate genes or proteins. However, unlike many of the above species, *P.ludlowii* seeds within the same carpel are not completely aborted. Yet aborted and normal seeds coexist, leading us to speculate that unique regulatory mechanisms exist in these plants.

To reveal the regulatory mechanism of ovule abortion in *P.ludlowii*, we sequenced the transcriptome using high-throughput sequencing technology based on morphological observations. We constructed the first transcript catalog of *P.ludlowii* seeds. We also identified and analyzed differentially expressed genes in normal and aborted seeds through multiple pathways based on the transcriptome sequencing results, which provides valuable insights into the molecular regulatory mechanisms of ovule abortion in woody perennials.

## Materials and methods

### Acquisition of experimental materials

**Fig. 2:**
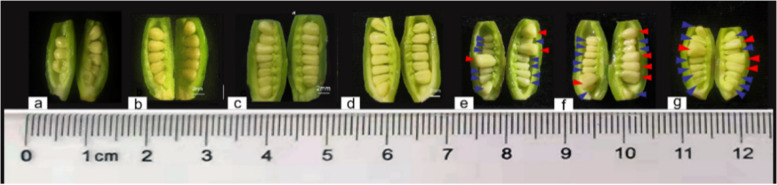
Test materials: **a**-**d**: ovules at 9, 10, 11, 12 DAF; **e**–**g**: normal and aborted ovules at 13, 15, 17 DAF. Normal and aborted ovules are indicated by red and blue arrows, respectively

*Paeonia* *ludlowii* materials were collected from three individual flowering trees as the methods used by our laboratory before [34]. The materials we obtained were all from the Ex-situ Conservation Centre of Chinese *Paeoniceae* Wild Species (Luanchuan County, Henan Province, China) (111°21′36.15″E, 33°56′4.99″N, 1 408 m altitude). The plant materials used in our experiments were collected in compliance with relevant institutional, national and international guidelines and legislation. Li Qingdao, former Secretary of the Party Committee and professor level senior engineer of the Sui and Tang Dynasties City Ruins Botanical Garden in Luoyang, Henan Province, China undertook the formal identification of the plant material used in our study. And We have sent the voucher specimen of this material to the Museum of Beijing Forestry University, the herbarium collection number is HNLC052101. Three to five carpels were periodically dissected to observe ovule development beginning when the flowers opened on 2020 21 May. The ovules were also selected from the same flowering tree from 25 May 2020 to 15 June 2020, and the carpels were collected at 9 d after flowering (DAF), 10 DAF, 11 DAF, 12 DAF, 13 DAF, 15 DAF, and 17 DAF. The ovules were removed after rapid peeling and photographic recording (Fig. 2). Ovule samples were named Pl-1 (ovules at 9 DAF), Pl-2 (ovules at 10 DAF), Pl-3 (ovules at 11 DAF), Pl-4 (ovules at 12 DAF), Pl-5N (normal ovules at 13 DAF), Pl-5A (aborted ovules at 13 DAF), Pl-6N (normal ovules at 15 DAF), Pl-6A (aborted ovules at 15 DAF), Pl-7N (normal ovules at 17 DAF), and Pl-7A (aborted ovules at 17 DAF). All samples were quickly frozen in liquid nitrogen and stored at − 80 °C for subsequent experiments.

### Ultrastructure observation

Samples were prepared according to the methods used by Donghui Wang (2017) [35]. After properly trimming the embedding samples, they were sectioned using an ultrathin sectioning machine (Leica EM UC7; Wetzlar, Germany), stained with a small droplet staining method 2% UO2 acetate for 1 h, rinsed 2–3 times with ultrapure water, and blotted dry with filter paper. The same small droplet staining method was used for lead citrate staining for 5–10 min, after which it was rinsed 2–3 times with ultrapure water, dried naturally, and observed by TEM (JEOL JEM-1010, Japan).

### RNA extraction, cDNA library construction, and transcriptome sequencing

Our laboratory's preliminary experimental results showed structural differences between normal and abortion ovules from 9 DAF [36]. Therefore, we selected the ovule samples from the 9th to 17th DAF for transcriptome sequencing. Total RNA was extracted using an RNA prep Pure Plant Kit (Polysaccharides& Polyphenolics-rich) (Tiangen, Beijing, China). Three biological replicates were prepared for each sample. RNA purity was checked using a Nano Photometer® spectrophotometer (IMPLEN, CA, USA), and OD_260_/OD_280_ readings between 1.8 and 2.1. After RNA samples were validated, RNA-seq libraries were prepared using NEBNext® Ultra™ RNA Library Prep Kit for Illumina® (NEB, USA) following manufacturer’s recommendations and index codes were added to attribute sequences to each sample and sequenced at Novogene Co. Ltd (Beijing, China). Transcriptome sequencing was conducted on the Illumina HiSeq^TM^2000 high throughput sequencing platform.

### Transcriptome assembly and functional gene annotation

There is currently no reference genome for *P.ludlowii*, so we sequenced the non-reference transcriptome. The transcriptome data of *P. ludlowii* have been uploaded to NCBI (http://www.ncbi.nlm.nih.gov/) (PRJNA818047). Clean data is obtained by removing reads containing aptamers after sequencing data is complete (clean reads), ploy-N, and low-quality reads from the raw data. At the same time, Q20, Q30, GC-content, and sequence duplication levels of the clean data were calculated. All downstream analyses were based on clean data with high quality. We analyzed the variation between each group of three samples and added a PCA plot based on the expression of all single genes in each sample (Additional Fig. 1). We spliced clean reads from sequencing into transcripts using Trinity (v2.4.0) [37] and performed hierarchical clustering with Corset (version 4.6) program transcripts [38]. Each cluster was defined as “Gene”. Finally, the longest transcript in each cluster was as the “unigene” of the gene for subsequent analysis. We used BUSCO (Benchmarking Universal Single-Copy Orthologs) software to evaluate the splicing quality of the data obtained from the splicing and evaluate the splicing quality, also evaluate the accuracy and integrity of the splicing results according to the proportion and integrity of the comparison (Additional Fig. 2). Gene functions were annotated based on the following databases and tools. Nr (NCBI non-redundant protein sequences, diamond v0.8.22); Nt (NCBI non-redundant nucleotide sequences, NCBI blast 2.2.28 +); Pfam (Protein family, HMMER 3.0 package); KOG/COG (Clusters of Orthologous Groups of proteins, diamond (v0.8.22)); Swiss-Prot (A manually annotated and reviewed protein sequence database, diamond (v0.8.22)); KO (KEGG Ortholog database, KAAS (r140224)); GO (Gene Ontology, blast2go (b2g4pipe_v2.5)).

Take the unigenes obtained from Trinity splicing as the reference sequence (Ref), and map the clean reads of each sample to Ref. The comparison process was completed by calling bowtie2 with RSEM software [39], and the used bowtie2 parameter is the default parameter (mismatch 0). The number of reads matched to a gene is called read count. Then, we used FPKM (expected number of Fragments Per Kilobase of translation sequence per Millions base pairs sequenced) to convert the read count to FPKM [40]. The FPKM calculation formula is:

$$\mathrm{FPKM}=\frac{\mathrm{mapped}\;\mathrm{fragments}\;\mathrm{of}\;\mathrm{transcript}}{\mathrm{Total}\;\mathrm{Count}\;\mathrm{of}\;\mathrm{mapped}\;\mathrm{fragments}\;(\mathrm{Millions})\;\times\;\mathrm{Length}\;\mathrm{of}\;\mathrm{transcript}\;(\mathrm{kb})}$$

### Differential expression analysis and enrichment analysis

Differential expression analysis was performed using the DESeq R package (1.10.1). The screening conditions were: False Discovery Rate (FDR) adjusted p-value < 0.05 and | log2FC |> 1. We then used GO seq [41] and KOBAS software [42] to perform GO (Gene Ontology) enrichment analysis and KEGG (Kyoto Encyclopedia of Genes and Genomes) [43] pathway enrichment analysis of the DEGs (differentially expressed genes). Based on GO and KEGG enrichment analysis of DEGs, structural genes related to plant nutrition, plant hormone signal transduction, programmed cell death, ovule development and abortion were screened from differential genes, and the expression pattern of structural genes was analyzed.

### QRT-PCR validation

Liu et al. demonstrated that *RPS9* was the most suitable internal reference gene for peony seeds at different developmental stages [44]. Therefore, we used the peony *RPS9* gene as an internal reference gene in this paper. The primer sequences used in this paper are shown in Additional file 1. The qRT-PCR reactions were performed on a qTOWER2.2 fluorescent qPCR instrument (jena, Germany), according to the instructions included in the NovoStart® SYBR qPCR SuperMix Kit (Novoprotein). A negative control was set for each gene during the reaction, and three biological replicates were designed for all experiments. Finally, the relative expression of the target gene was calculated according to the 2^−ΔΔCT^ algorithm [45].

## Results

### Structural differences between normal and aborted ovules in the early stages of embryonic development in *Paeonia ludlowii*

**Fig. 3:**
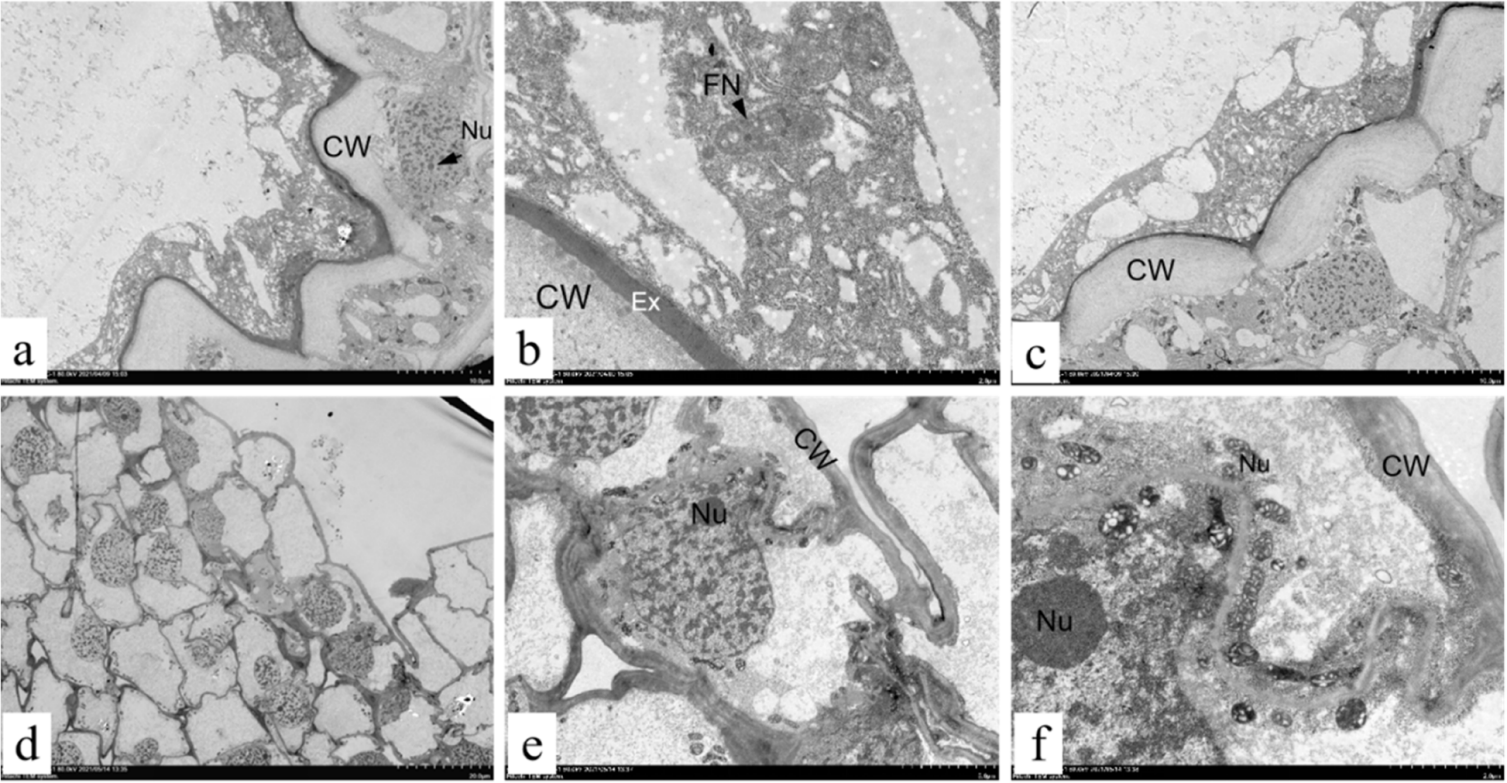
Ultrastructure of normal ovules and aborted ovules 15 days after natural pollination: CW: cell wall; EX: exudate; FN: free nuclear; Nu: nucleolus; **a**-**c**: The endosperm free nucleus of the normal ovule is intact; **d**-**f**: The free nuclear endosperm of aborted ovules is completely degraded, and the inner integument cells are significantly different from normal ovules

After ultrastructural observation of ovules with significantly different external morphology 15 DAF after natural pollination, we found significant differences in their ultrastructures. In normal ovules, the free nucleus of the endosperm was intact (Fig. 3a), the nucleolus and chromatin structures could be distinguished, and a large amount of cytoplasm was distributed around the free nucleus (Fig. 3b). In the cells of the inner beads, the nucleolus, nuclear membrane, chromatin structures could be distinguished, and a large amount of secretion was outside the cell wall. In contrast, a large number of mitochondria was distributed along the cell wall (Fig. 3c). In the aborted ovules, the endosperm-free nucleus and cytoplasm were degraded. Only some traces remained, a large number of inner beads near the embryo sac were reduced in cell contents (Fig. 3d), the plasma membrane was wrinkled, the cell wall was thinner and more deeply stained than in normal ovules, almost no secretion was observed outside the cell wall, and chromatin in some of the nuclei tended to be marginalized (Fig. 3e, f). Similar to the characteristics of programmed cell death (PCD) in some plant cells [46–48], this suggests that the PCD process may initiated early in *P.ludlowii* ovules, and premature PCD could be a cause of their early embryo and endosperm abortion.

### Transcriptome sequencing results of *Paeonia ludlowii*

The data was filtered and screened to obtain high-quality clean reads for subsequent analysis. As a result, 6.0 Gb of clean reads were obtained for each sample: the GC content of reads in the samples ranged from 41.36% ~ to 44.25%, and the percentage of Q30 bases exceeded 92.19% for all samples (Additional file 2).

Splicing the clean reads with Trinity yielded 256,789 transcripts with an average length of 1,238 bp and an N50 value of 1,881. The longest transcript in each gene was considered a representative of that gene, which is called a unigene. In total, 114,350 unigenes were obtained. All unigenes obtained by trinity splicing were compared with seven public databases, and 50,323 unigenes were annotated.

### Nr function annotation for unigenes

**Fig. 4:**
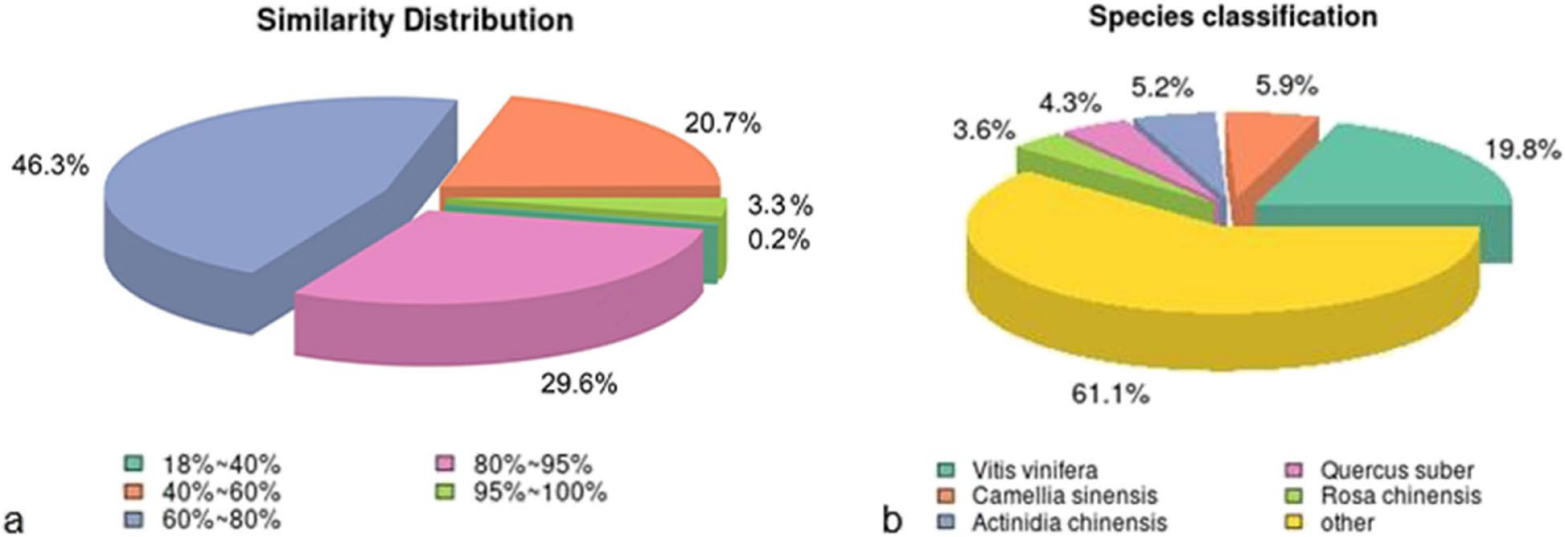
Nr Database comparison chart: **a** The similarity distribution map of unigenes compared with other species in the Nr annotation results; **b** Species distribution map on Nr library comparison

Based on the analysis of the annotation results of unigenes in Nr, it was found that 3.3% of the sequences of unigenes shared more than 95% identity with sequences of other species, 29.6% had 80%-95% identity of other species, and 46.3% had 60%-80% identity of other species (Fig. 4a). The function of Nr was annotated among the unigenes by diamond software (v0.8.22) with parameters set to e^−value^ = 1e^−5^, Nr function was annotated among the unigeneswith 7995 unigene sequences that were highly homologous with those of *Vitis vinifera*, accounting for 19.8% of the total, followed by *Camellia sinensis* (5.9%), *Actinidia chinensis* (5.2%), *Quercus suber* (4.3%), and *Rosa chinensis* (3.6%) (Fig. 4b).

### Screening for differentially expressed genes (DEGs)

**Fig. 5:**
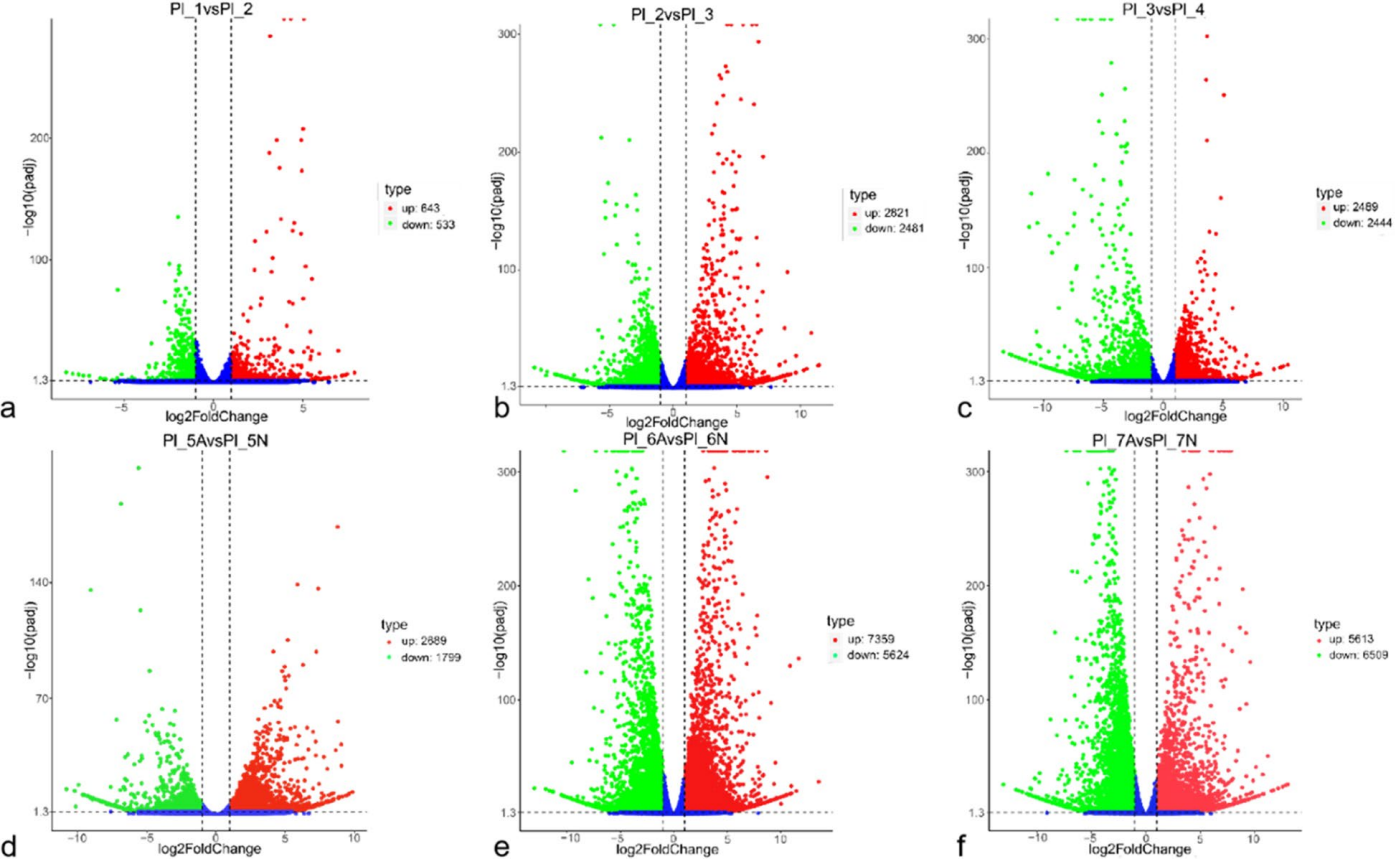
DEG volcano plot analysis of *Paeonia ludlowii*: **a** Pl_1 vs. Pl_2; **b** Pl_2 vs. Pl_3; **c** Pl_3 vs. Pl_4; **d** Pl_5A vs. Pl_5N; **e** Pl_6A vs. Pl_6N; **f** Pl_7A vs. Pl_7N. Red indicates upregulation, and green represents downregulation

**Fig. 6:**
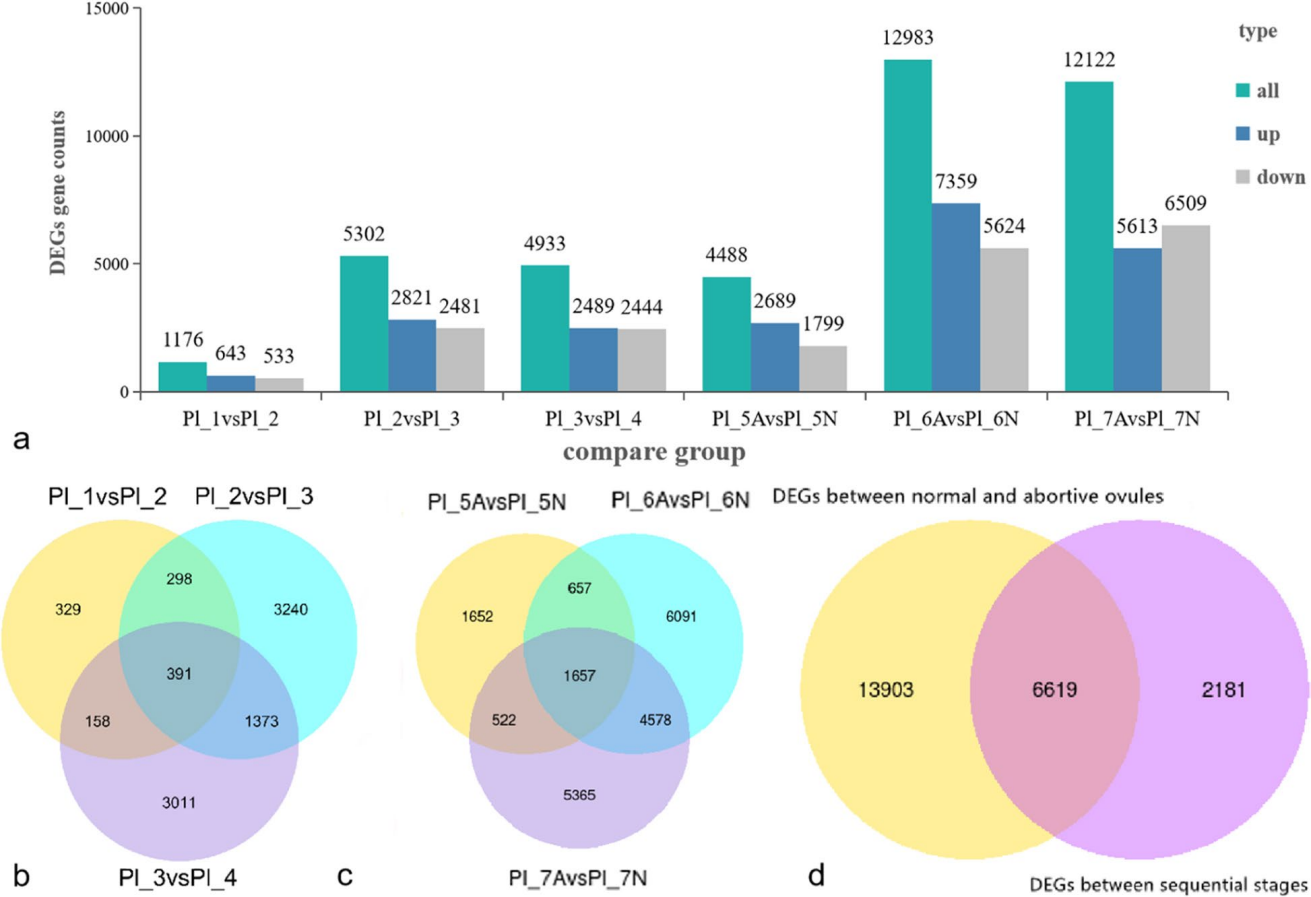
DEGs of *Paeonia ludlowii*: **a** The number of DEGs; **b** Venn diagram of DEGs during the adjacent period of early abortion; **c** Venn diagram of DEGs between early aborted ovules (A) and normal ovules (N); **d** Venn diagrams of all DEGs

Since Paraffin sections revealed that cell degeneration already occurred inside in the embryo sac of aborted ovules at 9 DAF, so DEGs were screened between samples from four adjacent ovule developmental periods at 9 DAF (Pl_1), 10 DAF (Pl_2), 11 DAF (Pl_3), and 12 DAF (Pl_4) (Pl_1 vs. Pl_2, Pl_2 vs. Pl_3, Pl_3 vs. Pl_4) (Figs. 5a-c, Fig. 6a). Removing duplicate genes yielded a total of 8,800 DEGs (Fig. 6b). Of them, the fewest DEGs existed between (Pl_1 vs. Pl_2), with only 1,176, including 643 up-regulated genes and 533 down-regulated genes (Fig. 5a); the most DEGs existed between (Pl_2 vs. Pl_3) 2,821 up-regulated expressions and 2,481 down-regulated expressions (Fig. 5b); the rest of (Pl_3 vs. Pl_4), with 2,489 up-regulated genes and 2,444 down-regulated genes (Fig. 5c).

The external morphology of the ovules of *P.ludlowii* begins to show small differences in the volume of normal and aborted ovules at 12 DAF, a clear distinction between normal and aborted ovules was explored at 13 DAF. To understand the mechanism of aborted ovule formation in *P.ludlowii*, the transcriptome data of aborted ovules (A) and normal ovules (N) of *P.ludlowii* were analyzed for differences between sample groups (Pl_5A vs. Pl_5N, Pl_6A vs. Pl_6N, Pl_7A vs. Pl_7N) (Figs. 5d-f, Fig. 6a). After the comparison we removed the genes in the overlapping part of the Venn diagram, so that the duplicates only appear once, and then 20,522 DEGs were finally obtained between A and N (Fig. 6c). Among them, 4,488 DEGs, 2,689 up-regulated genes, and 1,799 down-regulated genes were obtained between A and N at Pl_5 (Fig. 5d); 7,359 up-regulated genes and 5,624 down-regulated genes were obtained between A and N at Pl_6 (Fig. 5e); 12,122 DEGs, containing 5,613 up-regulated genes and 6,509 down-regulated genes, were screened at Pl_7 (Fig. 5f).

Notably, 6,619 of the 8,800 DEGs in the adjacent period duplicated DEGs between A and N. To focus the analysis on genes that are truly differentially expressed, 22,703 DEGs were available for subsequent analysis after removing duplicates (Fig. 6d).

### GO function annotation for differentially expressed genes

**Fig. 7:**
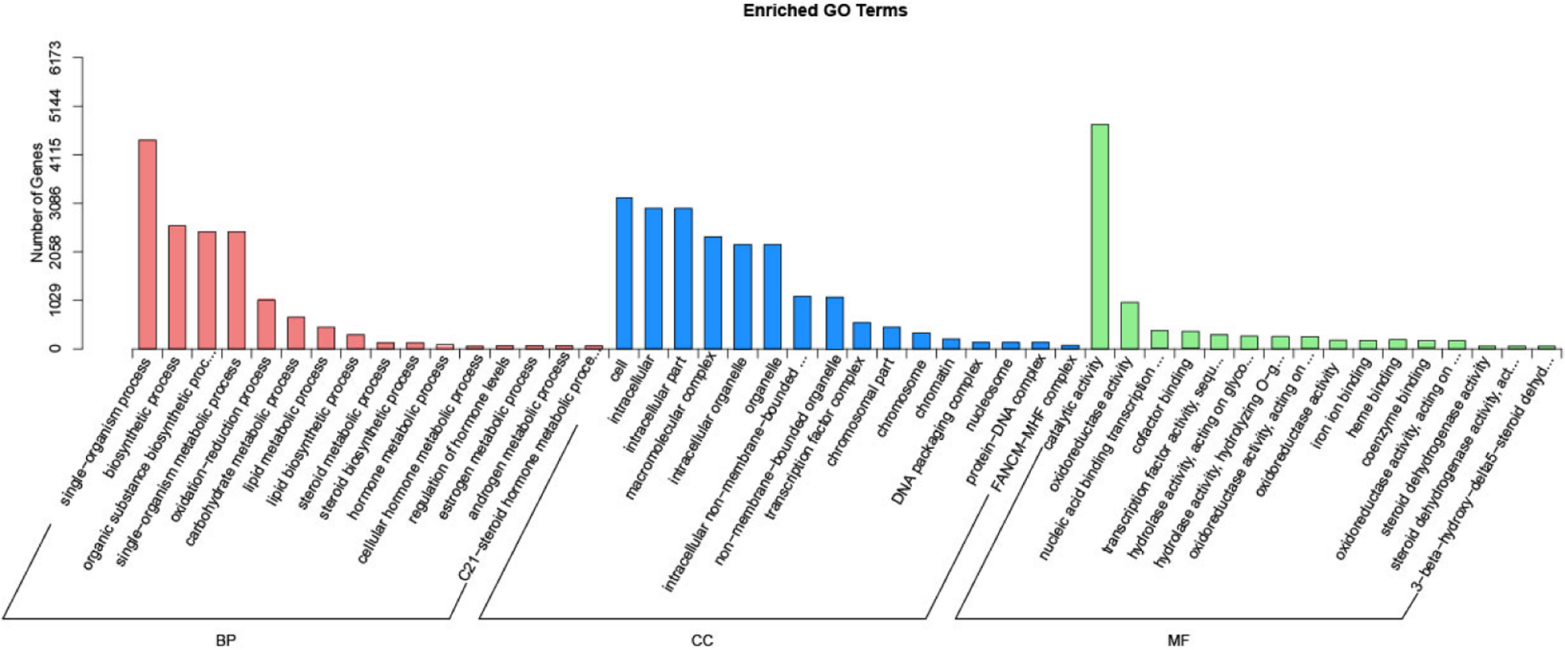
GO enrichment diagrams of DEGs (Top 48): Unigenes were annotated in three categories: biological processes, cellular components, and molecular functions

A total of 10,288 DEGs were annotated into 133 secondary terms of 3 major categories: biological process (BP), cellular component (CC), and molecular function (MF). Secondary classification and enrichment analysis of the 22,703 DEGs were performed together (Fig. 7, Additional file 3). The BP processes with the highest enrichment and high DEGs include the metabolic, single-organism, and biosynthetic processes. DEGs are primarily associated with the cell, cell parts, and intracellular elements relating to CC. The DEGs involved in MF, on the other hand, were mainly associated with catalytic activity, followed by oxidoreductase activity and nucleic acid binding transcription factor activity. These results suggest that metabolic and cellular differences during development could contribute to ovule abortion.

### KEGG function annotation for differentially expressed genes

**Fig. 8:**
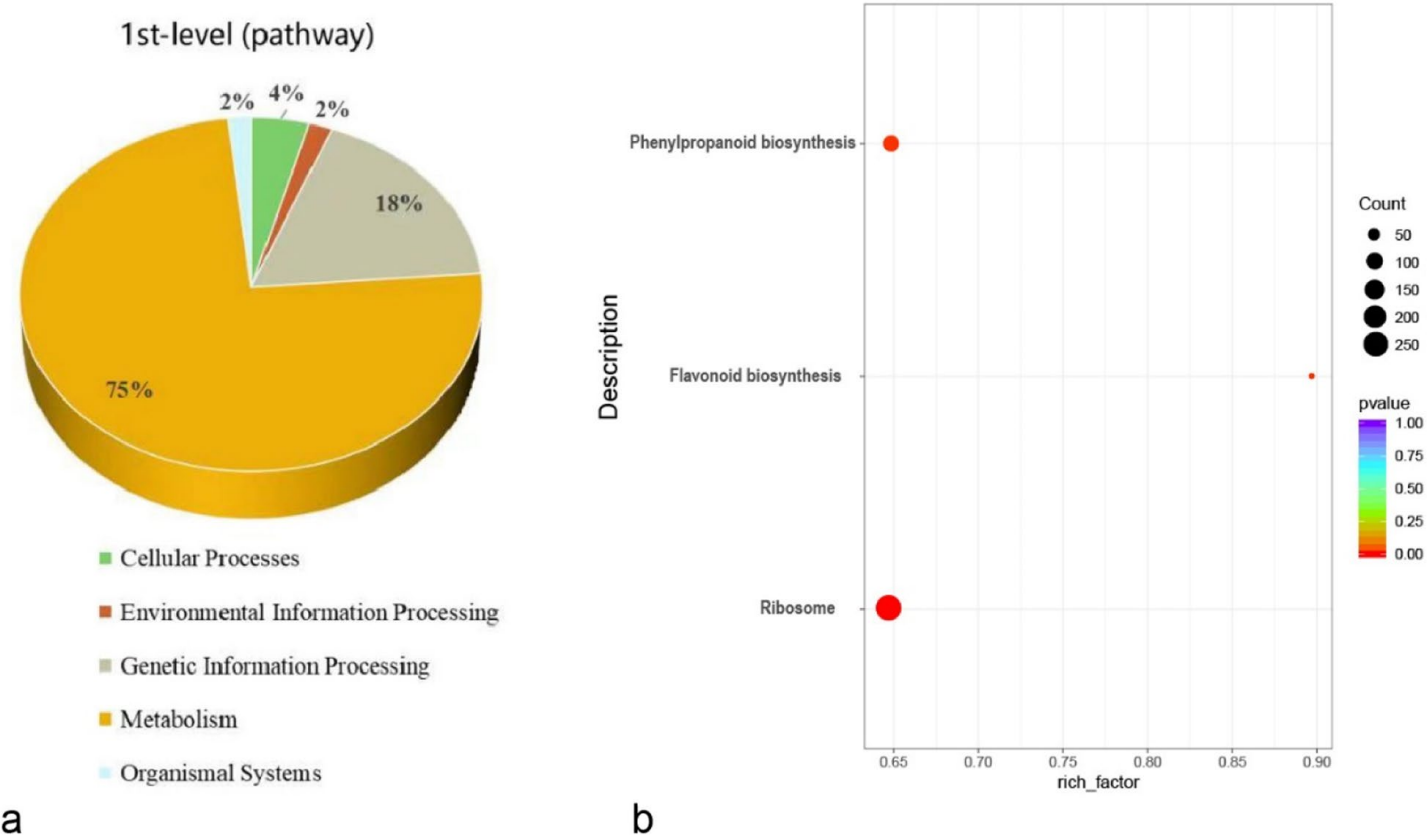
KEGG Enrichment and functional classification of DEGs in *Paeonia ludlowii*: **a** DEGs involved in KEGG pathways; **b** KEGG enrichment analysis. The Y-axis indicates the pathway name, the X-axis shows the richness factor, the size of the points represents the number of DEGs in the pathway, and the color of the points corresponds to different *p*-value ranges

To identify the major biometabolic pathways and signal transduction pathways involved in DEGs during ovule abortion in *P.ludlowii*, the identified DEGs were enriched for KEGG metabolic pathway functions [43]. A total of 4258 DEGs were annotated into 118 pathways belonging to five categories (Additional file 4). Of them, many DEGs were enriched to pathways related to genetic information processing, such as ribosomes. For biosynthetic pathways, DEGs are primarily annotated to phenylalanine biosynthesis and flavonoid biosynthesis (Fig. 8). These results indicate that normal and aborted ovules differ significantly in tissue development and nutrient accumulation during development. Additionally, plant hormone signal transduction, plant-pathogen interaction endocytosis, and phagosome pathways are also enriched in many DEGs, which are thought to play a key role in regulating plant seed abortion (Additional file 4).

### Expression of DEGs associated with carbohydrate metabolism during ovule abortion

**Fig. 9:**
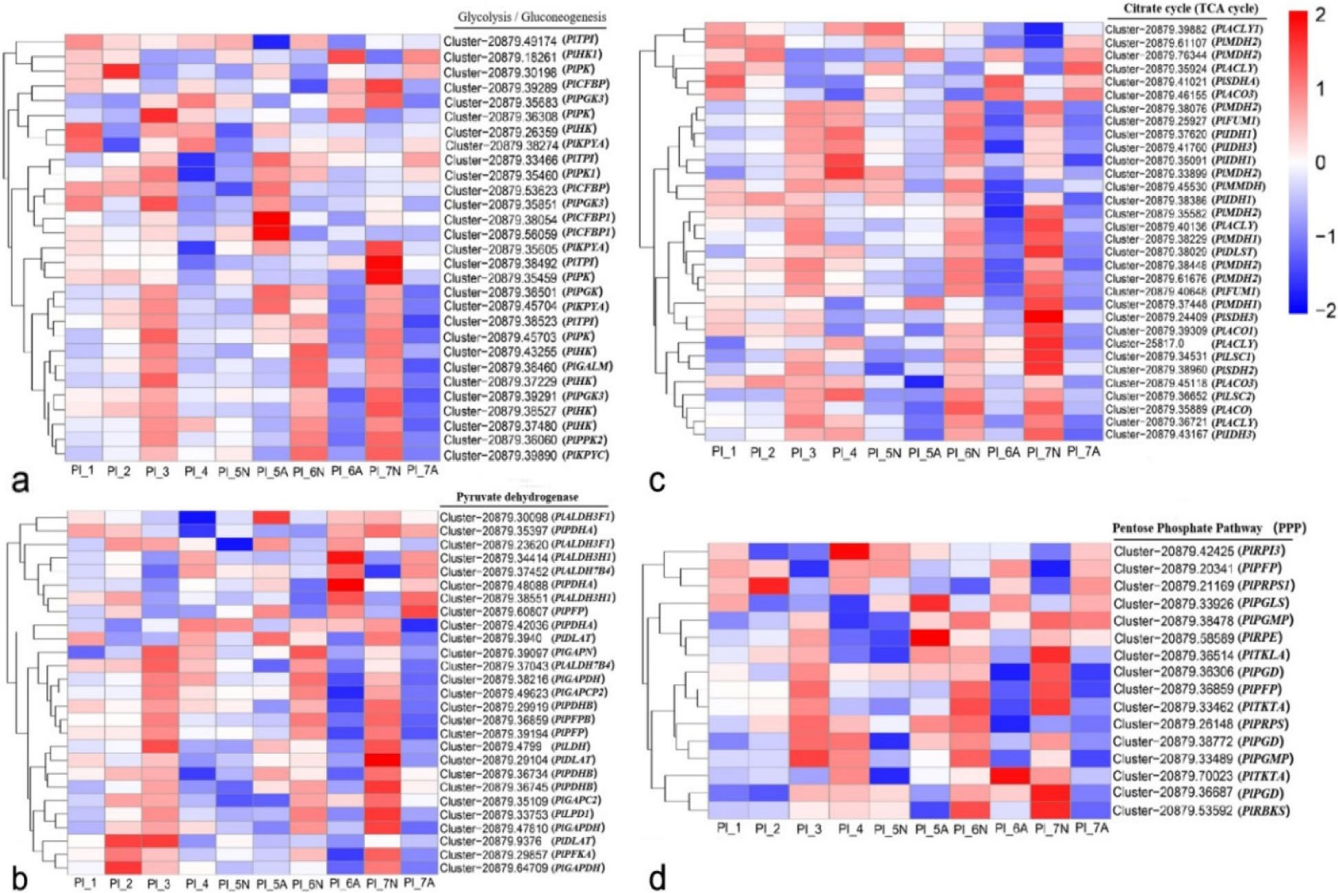
Expression patterns of DEGs associated with carbohydrate metabolism of *Paeonia ludlowii.* **a** Expression pattern of DEGs in glycolysis pathway; **b** Expression pattern of DEGs in pyruvate dehydrogenase; **c** Expression pattern of DEGs in citrate cycle; **d** Expression pattern of DEGs in pentose phosphate pathway. The unigene ID is on the left of each step. The Heatmap was constructed based on log_10_ (FPKM)

Glycolysis/gluconeogenesis, citrate cycle (TCA cycle), and pentose phosphate pathway (PPP) are the three major pathways of plant energy metabolism [49]. Analysis of DEGs revealed that many DEGs encode the three pathways in normal ovules and aborted ovules (Fig. 9, Additional file 5). The process includes genes encoding key glycolytic pathway enzymes: hexokinase genes *PlHK* and *PlHK3*, pyruvate kinase genes *PlPK*, *PlKPYA*, *PlPKP2,* and *PlPKP4* (Fig. 9a). The pyruvate dehydrogenase E1 gene *PlPDHB*, the pyruvate dehydrogenase E2 gene *PlDLAT*, and the 6-pyruvate dehydrogenase genes *PlPFKA* and *PlALDH7B4*, are responsible for the entry of pyruvate into the TCA cycle (Fig. 9b). Genes encoding key enzymes involved in the TCA cycle include pyruvate dehydrogenase citrate (Pro-S), cleavage enzyme gene *PlACLY*, aconitase genes *PlACO* and *PlACO1*, and malate dehydrogenase genes *PlMDH1* and *PlMDH2* (Fig. 9c). The key enzyme genes encoding the pentose phosphate pathway include the 6-phosphogluconate dehydrogenase gene *PlPGD* and the transketolase gene *PlTKLA* (Fig. 9d). Analysis of DEGs involved in the three major pathways demonstrates that the transcript levels of some unigenes continued to increase with ovule development and then decreased significantly from Pl_4. In the subsequent developmental period, the unigenes were mostly up-regulated in normal ovules and down-regulated in aborted ovules. This indicates that plant energy metabolism levels decreased during ovule abortion. Meanwhile, we identified unigenes involved in the three major pathways, including two glycolysis/gluconeogenesis unigenes *PlCTPI* (Cluster-20879.33466), *PlHK1*(Cluster-20879.18261) and one pentose phosphate pathway (PPP) unigene, *PlTKTA* (Cluster-20879.70023), which were also up-regulated in aborted ovules during Pl_5-Pl_7. The low expression of these genes in aborted ovules of *P. ludlowii* indicates that the carbohydrate synthesis pathway of aborted ovules is affected, and we hypothesize that the nutrient and energy supply of aborted ovules would be severely impaired for this reason, confirming the low viability of aborted ovules [34].

### Expression of DEGs associated with phytohormone signaling during ovule abortion

**Fig. 10:**
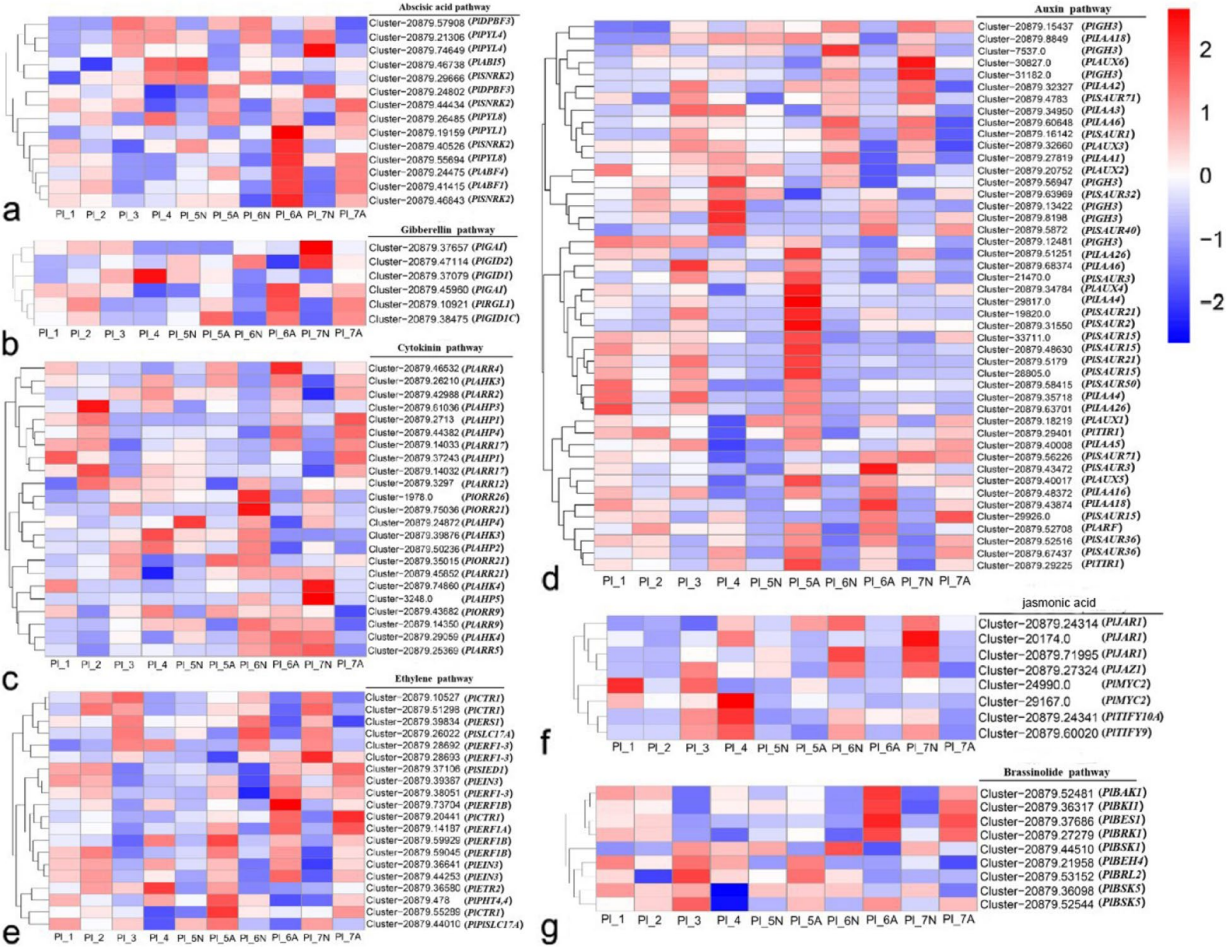
Expression patterns of DEGs associated with phytohormones signaling of *Paeonia ludlowii.* **a** DEG expression pattern of genes related to ABA; **b** Expression pattern of DEGs in GA pathway; **c** Expression pattern of DEGs in CTK pathway; **d** Expression pattern of DEGs in IAA pathway. **e** Expression pattern of DEGs in ETH pathway. **f** Expression pattern of DEGs in JA pathway. **g** Expression pattern of DEGs in BR pathway. The unigene ID is on the left of each step, and the sample name is on the bottom. The Heatmap was constructed based on log_10_ (FPKM)

During phytohormone signaling in aborted ovules of *P.ludlowii*, there are changes in the expression of genes related to multiple phytohormone signaling pathways. We focused on the differential expression of genes related to seven phytohormones: abscisic acid (ABA), gibberellin (GA), cytokinin (CTK), auxin (IAA), ethylene (ETH), brassinosteroids (BR), and jasmonic acid (JA) (Fig. 10). These include the ABF transcription factor family associated with the ABA signaling pathway, the GA receptor *GID1* gene, the CTK receptor *AHK* gene, the CTK response factor ARR protein, the CTK regulatory protein AHP, the IAA early response gene family SAUR (small auxin up RNA), the IAA input vector *AUX/IAA* gene, the IAA response gene *GH3*, the JA receptor JAZ protein, *JAR* genes involved in JA biosynthesis, and JA signal response factor *MYC2*. Homologs of the BR response transcription factor *BES1* were up-regulated in normal ovules during the Pl_1-Pl_4 and Pl_5-Pl_7 periods and down-regulated in aborted ovules. In addition, the *EIN3* and *ERF1* genes related to ETH signal response factor, the ETH receptor *ERS* gene, BR receptor kinase BRI1, and the BR signal kinase gene *BSK* were down-regulated in normal ovules and up-regulated in aborted ovules during the ovule development period. Many genes homologous to phytohormones were involved, indicating that the phytohormone key-related genes in vivo play an essential role in ovule abortion in *P.ludlowii*.

Several phytohormone-related genes were under-expressed to undetected levels during aborted ovules, relevant information is displayed in Additional file 6. The expression profiles of genes encoding differentially expressed proteins related to the synthesis, response, and transport of different phytohormones in *P.ludlowii* showed a diverse distribution after the initiation of abortion.

### Differential analysis of apoptosis and programmed cell death (PCD)-related genes in ovules

**Fig. 11:**
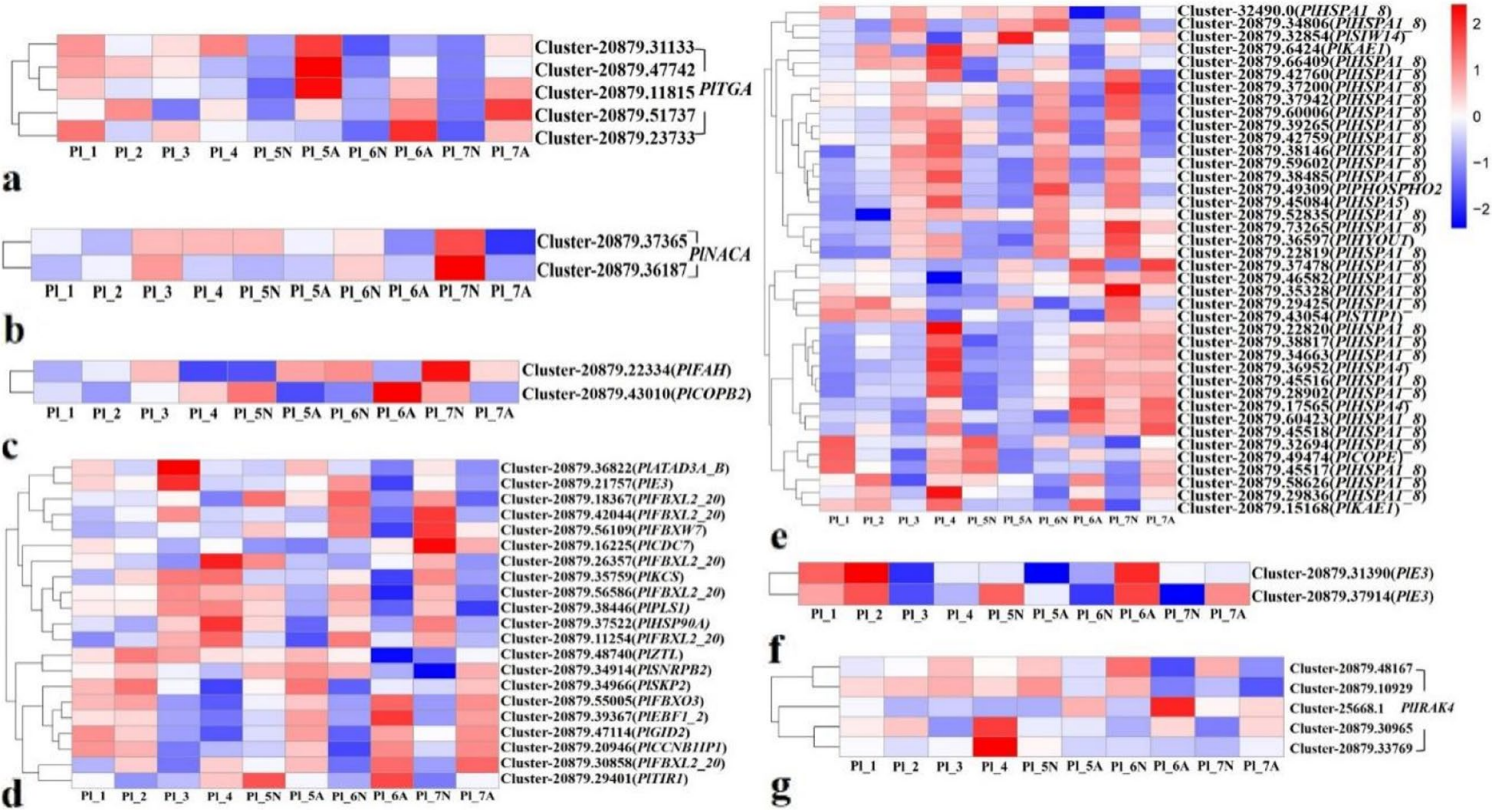
DEGs involved in PCD of *Paeonia ludlowii*: **a** DEGs expression pattern of *TGA* genes; **b** DEGs expression pattern of *NAC* genes; **c** DEGs expression pattern of *BAG* genes; **d** DEGs expression pattern of F-box genes; **e** DEGs expression pattern of *HSP70* genes; **f** DEGs expression pattern of aspartic proteinase genes; **g** DEGs expression pattern of cysteine-rich receptor-like protein kinase genes; The left side of each step in the expression pattern diagram is the gene ID, and the bottom is the sample name. Heatmap was constructed based on log_10_ (FPKM)

Among the DEGs of *P.ludlowii* ovules, several genes are involved in the regulation of apoptosis., These include genes encoding the F-box protein family and the molecular chaperone HSP70/HSc70. Additionally, a small number of genes related to *TGA*, *NAC*, cysteine-rich receptor-like protein kinase, *BAG*, and aspartic proteinase are related to programmed cell death in *P.ludlowii.* The expression level of *TGA* genes in aborted ovules is higher than that in normal ovules during Pl_5-Pl_7. On the contrary, the *NAC* gene was highly expressed in normal ovules during the Pl_5-Pl_7 period. Aspartic proteinase genes are highly expressed in aborted ovules during Pl_6-Pl_7. Finally, we focused on *HSP* and F-box-related genes; their expression patterns in normal ovules and aborted ovules were diverse during the developmental process of *P.ludlowii*. Therefore, we speculated that *HSP* and F-box-related genes played various roles in programmed ovule death in *P.ludlowii* (Fig. 11, Additional file 7).

### Analysis of DEGs related to ovule development and abortion

**Fig. 12:**
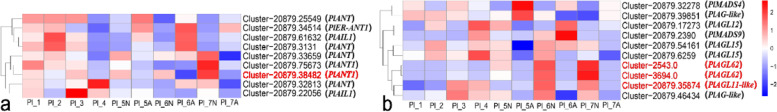
Expression of DEGs involved in the development ovule of *Arabidopsis* in *Paeonia ludlowii*. **a** Expression of *ANT* and *AIL* relevant DEGs involved in the development ovule of *Arabidopsis* in *Paeonia ludlowii*; **b** Expression of *MADS*, *AG* and *AGL* relevant DEGs involved in the development ovule of *Arabidopsis* in *Paeonia ludlowii*. The unigenes shown in red are the key genes mentioned in the article

Key regulators of *Arabidopsis* ovule development include *AG*, *INO*, *SPL*, *ANT*, and *BELI* [50–52]. A total of 19 DEGs homologous to *AG* and *ANT* were obtained from the *P.ludlowii* ovule samples (Fig. 12). After the ovules of *P.ludlowii* were aborted (Pl_5-Pl_7 period), one *ANT* homolog *PlANT1* (Cluster-20879.38482) and three *AG* homologs, *PIAGL11*-like (Cluster-20879.35874), *PlAGL62* (Cluster-2543.0), and *PlAGL62* (Cluster-3694.0) were consistently up-regulated in normal ovules during the Pl_5-Pl_7 period, down-regulated or not expressed in aborted ovules, and significantly more highly expressed in normal ovules than in aborted ones (Fig. 12, Additional file 8).

**Fig. 13:**
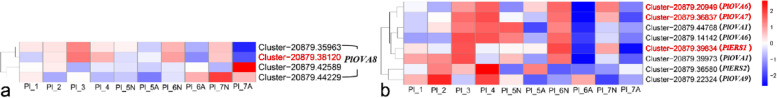
Expression of DEGs involved in ovule abortion of *Paeonia ludlowii*. **a** Expression of *OVA8* relevant DEGs involved in ovule abortion of *Paeonia ludlowii*; **b** Expression of *OVA6*, *OVA7*, *OVA1*, *OVA9* and *ERS* relevant DEGs involved in ovule abortion of *Paeonia ludlowii*. The unigenes shown in red are the key genes mentioned in the article

The *Arabidopsis* gene sequence database (https://www.Arabidopsis.org/) was searched for the keyword “ovule abortion” and produced nine genes related to ovule abortion. When these genes were knocked out, the ovule abortion rate of *Arabidopsis* mutants was significantly higher than that of wild plants [53] (Table 1). Using the above nine genes as a reference, combined with the expression of DEGs, GO, and KEGG enrichment results, 12 homologs were mined, including two *ERS* homologs *PlERS1* (Cluster-20879.39834), one *OVA6* homolog *PlOVA6* (Cluster-20879.20949), one *OVA7* homolog *PlOVA7* (Cluster-20879.36837). The expression of *PlOVA8* (Cluster-20879.38120) was more down-regulated in aborted ovules after the apparent defeat (Pl_5-Pl_7) and up-regulated in normal ovules. Additionally, the expression level in aborted ovules was significantly lower than in normal ovules (Fig. 13, Additional file 9).

**Table 1:** Genes associated with ovule abortion in *Arabidopsis*

Gene name	Other names	Gene Model	Phenotypes
*OVA7*	*ovule abortion 7, SRS, ATSRS, Seryl-tRNA synthetase, Seryl-tRNA synthetase*	At1g11870.2	Among heterozygous plants with *OVA7* knocked out, 29% of ovules aborted, compared with only 6% of wild-type ovules. (Berg et al., 2005) [53].
*OVA9*	*ovule abortion 9*	At1g25350.2	In heterozygous plants, 39% of ovules aborted compared to 6% for wild type siblings (Berg et al., 2005) [53].
*OVA6*	*ovule abortion 6, PRORS1, Atprors-org, Prolyl-tRNA Synthetase 1, Prolyl-tRNA Synthetase Organellar, Prors-org*	At5g52520.1	In heterozygous plants, 56% of ovules aborted, compared to 5% in wild-type siblings. (Berg et al*.*, 2005) [53].
*OVA5*	*ovule abortion 5, ATKRS-2, Arabidopsis thaliana lysyl-tRNA Synthetase 2*	At3g13490.1	In heterozygous plants with *OVA5* knocked out, 37% of ovules aborted, compared with only 4% of wild-type ovules (Berg et al*.*, 2005) [53].
*OVA4*	*ovule abortion 4*	At2g25840.2	Among heterozygous plants with *OVA4* knocked out, 26% of ovules aborted, compared with 7% in the wild type (Berg et al*.*, 2005) [53].
*OVA2*	*ovule abortion 2*	At5g49030.1	In heterozygous plants with *OVA2* knocked out, 42% of ovules aborted, compared with 11% in the wild type (Berg et al*.*, 2005) [53].
*OVA1*	*ovule abortion 1*	At3g55400.1	In heterozygous plants with *OVA1* knockout, 33% of ovules aborted, compared with 11% in the wild type (Berg et al*.*, 2005) [53].
*ERS*	*OVA3、OVULE ABORTION 3, ERS, ATERS, GLUTAMATE TRNA SYNTHETASE*	At5g64050.1	In heterozygous plants with *OVA3* knocked out, 26% of ovules aborted, compared with only 9% in the wild type (Berg et al*.*, 2005) [53].
*OVA8*	*ATNS1, NS1, ovule abortion 8, Asparaginyl-tRNA synthetase*	At4g17300.1	Among heterozygous plants with *OVA8* knocked out, 26% of ovules aborted, compared with 6% in the wild type (Berg et al*.*, 2005) [53].

**Fig. 14:**
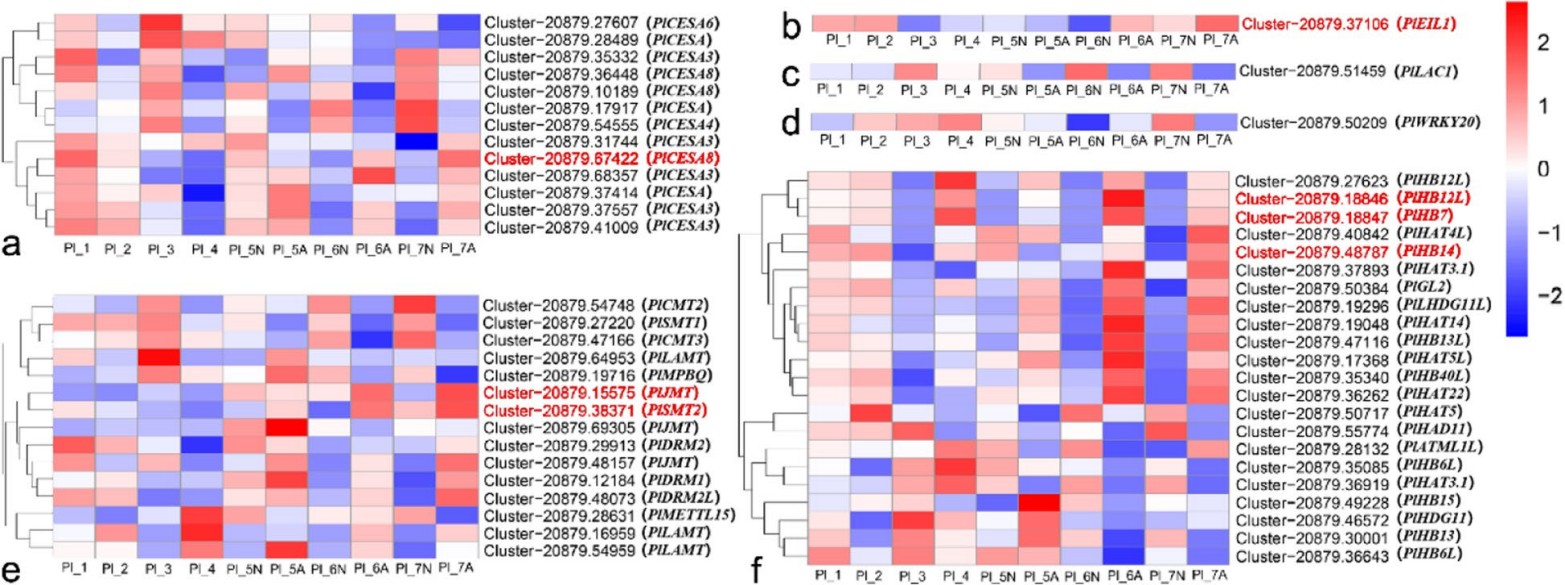
Expression of DEGs involved in aborted ovules of *Paeonia ludlowii*. **a** Expression of *CESA* relevant DEGs involved in involved in aborted ovules of *Paeonia ludlowii*; **b** Expression of *EIL* relevant DEGs involved in involved in aborted ovules of *Paeonia ludlowii*; **c** Expression of *LAC* relevant DEGs involved in involved in aborted ovules of *Paeonia ludlowii*; **d** Expression of *WRKY* relevant DEGs involved in involved in aborted ovules of *Paeonia ludlowii*; **e** Expression of *CMT*, *SMT*, *LAMT*, *MPBQ*, *IMT*, *DRM*, *METTLI* and *AMT* relevant DEGs involved in involved in aborted ovules of *Paeonia ludlowii*; **f** Expression of *HBI*, *HB*, *HAT*, *GL*, *HDGIIL*, *HAD* and *ATMLIL* relevant DEGs involved in involved in aborted ovules of *Paeonia ludlowii*. The unigenes shown in red are the key genes mentioned in the article

Additionally, a number of homologs were obtained in DEGs associated with ovule abortion in plants other than *Arabidopsis* (Fig. 14). These include a *CesA* homolog *PlCesA8* (Cluster-20879.67422), an HD-zip transcription factor family homolog *PlHB14* (Cluster-20879.48787), a *JMT* homolog *PlJMT* (Cluster-20879.15575), an *SMT2* homolog *PlSMT2* (Cluster-20879.38371), and an ETH signaling pathway transcription factor *EIL1* (Ethylene-insenstives3-like) homolog *PlEIL1* (Cluster-20879.37106), all of which were consistently up-regulated in aborted ovules at Pl_5-Pl_7. Its expression was significantly higher than that of normal ovules at the same time. Two HD-zip transcription factor family homologs, *PlHB12L* (Cluster-20879.18846) and *PlHB7* (Cluster-20879.18847) were also up-regulated in aborted ovules at Pl_5-Pl_7. At the same time, they were not expressed in normal ovules of the same period or were expressed at undetectable levels (Fig. 14**)**. We selected 15 key genes for future research into abortion of *P.ludlowii*: *ANT* homolog *PlANT1* (Cluster-20879.38482) and three *AG* homologs, *PIAGL11*-like (Cluster-20879.35874), *PlAGL62* (Cluster-2543.0), and *PlAGL62* (Cluster-3694.0), *ERS* homolog gene (Cluster-20879.39834), *OVA6* homolog *PlOVA6* (Cluster-20879.20949), *OVA7* homolog *PlOVA7* (Cluster-20879.36837), *OVA8* homolog *PlOVA8* (Cluster-20879.38120), *CesA* homolog *PlCesA8* (Cluster-20879.67422), the 3HD-zip transcription factor family homologs *PlHB14* (Cluster-20879.48787), *PlHB12L* (Cluster-20879.18846), and *PlHB7L* (Cluster-20879.18847), *JMT* homolog *PlJMT* (Cluster-20879.15575), *SMT2* homolog *PlSMT2* (Cluster-20879.38371), *PlEIL1* (Cluster-20879.37106). However, further investigation is required to establish whether the expression of these genes is related to ovule abortion in *P.ludlowii*.

### QRT-PCR validation of DEGs

**Fig. 15:**
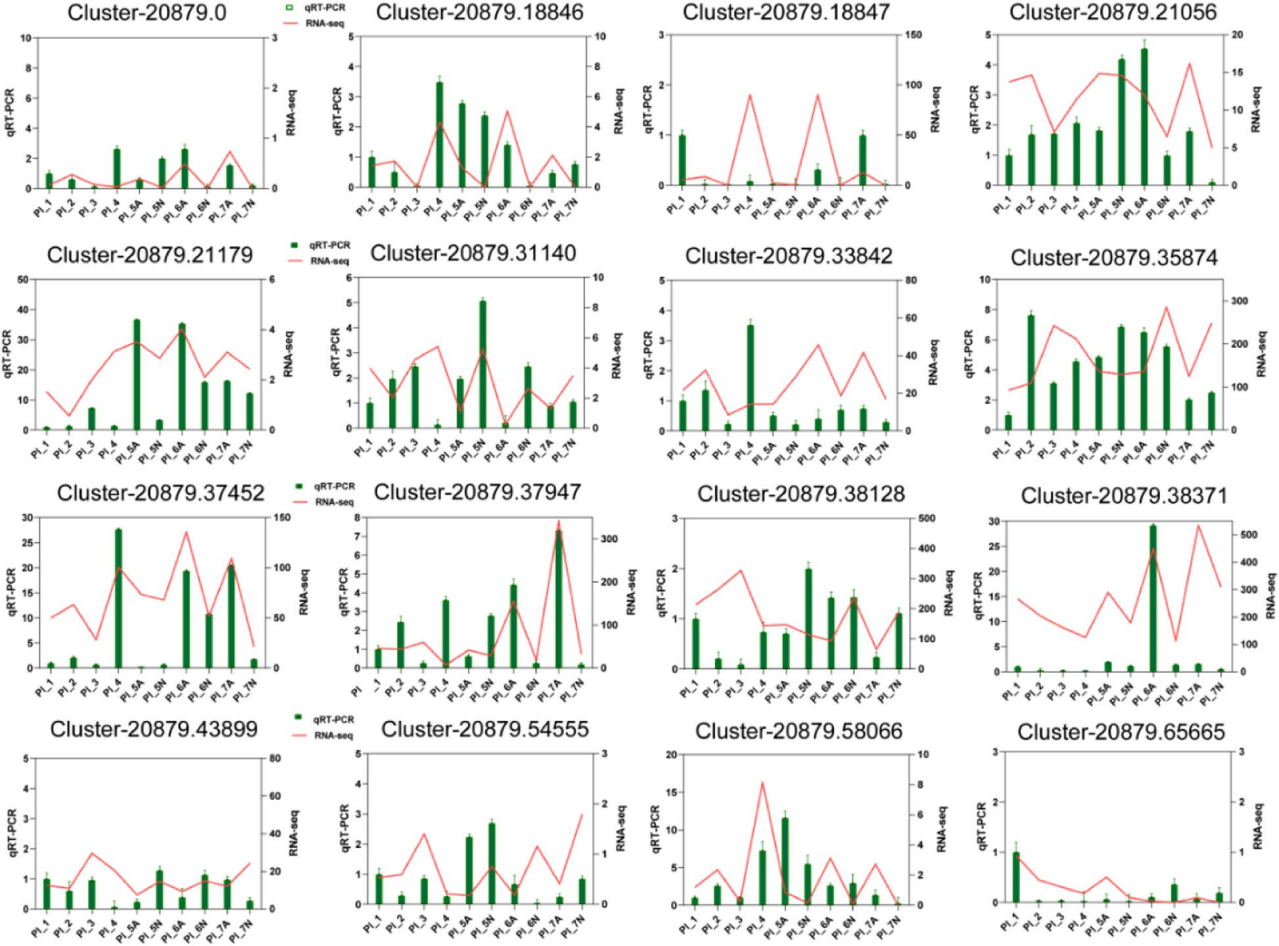
QRT-PCR validation of DEGs

To verify the credibility of the RNA-seq results and assess the cause of ovule abortion in *P.ludlowii*, 16 differential genes were selected for qRT-PCR analysis. The results demonstrated that the expression patterns of 16 of these genes in the qRT-PCR analysis were similar to those in the transcriptome sequencing (Fig. 15). This indicates that transcriptome data and qRT-PCR analysis data were accurate and reliable and that the genes screened in this study that play a key role in ovule abortion in *P.ludlowii* were reliable.

## Discussion

### Nutrients affect early seed abortion in *Paeonia ludlowii*

In *P.lutea*, a large number of DEGs between normal and aborted seeds were distributed in “metabolic process”, “binding” and “catalytic activity” [54]. Similar to the research results of *P.lutea*, during the seed development and abortion of *P.ludlowii*, a large number of DEGs were enriched in the pathways related to nutrient metabolism, including “biosynthetic process”. At the same time, a large number of DEGs were enriched in “phenylpropane biosynthesis” and other metabolic pathways. The expression of a large number of DEGs encoding TCA cycle and PPP pathway in Pl_1 ~ Pl_4 was down-regulated, and then in aborted seeds of Pl_ 5 ~ Pl_7 was down. The results showed that the abortion of *P.ludlowii* was related to the decomposition and accumulation of nutrients and energy metabolism. Previous studies found that the abortion of *P.ludlowii* was related to the catabolism and accumulation of nutrients [34]. In this paper, the low expression of genes related to the energy metabolism pathway in aborted seeds confirmed the low vitality of aborted seeds. It is speculated that the seeds accumulated a certain amount of energy before the abortion. Due to the gradual development in the later stage, the energy supply of the aborted seeds was insufficient, and the endosperm-free nucleus and cytoplasm degenerated, but some energy metabolism processes still existed.

### Phytohormones affect early seed abortion in *Paeonia ludlowii*

Phytohormones have an important regulatory role in ovule development [52]. Our results indicate that the expression profiles of genes encoding differentially expressed proteins of different phytohormones in ovules showed a diverse distribution, suggesting that the mechanisms of phytohormone regulation are complex. The ABA, BR, and ETH-related genes were heavily up-regulated in aborted ovules during Pl_5 to Pl_7 (Fig. 10). ABA levels are closely related to plant growth and development, and increases in ABA levels in aborted seeds of maize, date, and chrysanthemum have been described [55]. Also, the increase in ABA levels in lychee embryos alters the balance ratio of endogenous hormones and could affect embryo development, eventually leading to embryo abortion [56]. Brassinolide is a sterol plant hormone that participates in plant growth and various physiological reactions. It has been reported that BR can regulate plant growth and development by interacting with the ABA hormone. In this study, the contents of ABA and BR hormones increased in aborted ovules during the later stages of ovules development. Therefore, we speculate that ABA and BR interaction plays a vital role in regulating plant ovule abortion. Transcriptome analysis demonstrated that the *EIN3* and *ERF1* genes, which are related to ETH signal response factors and the ETH receptor *ERS* gene, were up-regulated in aborted ovules. Wanghui revealed the molecular mechanism of ethylene involved in regulating ovule abortion in seedless pears [57], demonstrating that ethylene-insensitive 3-like 1 (*EIL1*) is involved in regulating ovular abortion and senescence in seedless pears by directly affecting the transcription of CYSTEINE PROTEINASE 1(*Cysp1*), a cell senescence-related gene. Therefore, excessive accumulation of ethylene in the ovules of *P.ludlowii* could lead to premature senescence and death of the ovules. We believe that phytohormones play an essential role in the development of *P.ludlowii* ovules, and that regulating ovule development in *P.ludlowii* by external phytohormone application should be considered.

### Key regulatory genes for ovule development and abortion regulate early ovule abortion in *Paeonia ludlowii*

*ANT* is an AP2 subfamily transcription factor that encodes an *ANT* gene closely related to ovule primordium formation and ovule development and can regulate seed size [58–60]. *Arabidopsis* plants overexpressing *ANT* increased ovule size but did not change the ovule number [61]; the *Arabidopsis MEE45* (MATER NAL EFFECT EMBRYO ARREST45) transcription factor regulates seed size by inducing *ANT* expression [62]. The *PlANT1* (Cluster-20879.38482) gene was up-regulated in normal seeds after the ovules of *P.ludlowii* were aborted, and their expression was significantly higher than that of aborted ovules. This suggests that the *ANT* homolog *PlANT1* could play a key role in regulating ovule abortion in *P.ludlowii*.

*AG* genes regulate the abortion and development of floral organs, fruit and seed development [63]. In this chapter, three AG homologs, *PlAGL11*-like (Cluster-20879.35874), *PlAGL62* (Cluster-2543.0), and *PlAGL62* (Cluster-3694.0), were up-regulated in normal ovules relative to aborted ovules. Studies have demonstrated that the *AGL11* gene has an essential regulatory role in tomato ovule development and seed formation [64]. We found that the differential expression of *PlAGL11*-like between normal and aborted ovules indicates that *PlAGL11*-like plays a vital role in regulating seed abortion in *P.ludlowii*. During ovule development, *AGL62* is a major regulator of endosperm cellularization and is expressed only in the endosperm, where it inhibits endosperm cellularization at the syncytial stage and promotes nuclear proliferation [65]. In wild *Arabidopsis* seeds, *AGL62* is highly expressed at the endosperm cytosolic stage. It suddenly decreases before cellularization, while the deletion of *AGL62* leads to early endosperm cellularization, reduced nuclei, and lethal seed defects [66]. The high expression of two *PlAGL62* in normal ovules of *P.ludlowii* suggests that it could play the same regulatory role in regulating endosperm development in *P.ludlowii*, and that the low expression of *PlAGL62* in aborted ovules prevents the free nuclei of the endosperm from continuing to proliferate, leading to ovule abortion.

The *ERS*, *OVA6*, *OVA7*, and *OVA8* genes are important regulators of ovule development and abortion in *Arabidopsis*. In *Arabidopsis*, 26% of ovules of plants with knockout of the *ERS* and *OVA8* genes, respectively, were aborted compared with 6% of seeds of the wild type; 56% of ovules of plants with knockout of the *OVA6* gene were aborted compared with 5% of seeds of the wild type; and the ovule abortion rate of *OVA7* knockout plants was also much higher than that of wild-type plants [53]. This suggests that these genes play a positive regulatory role in ovule development, and their deletion will lead to abnormal ovule development and abortion. In this chapter, the *ERS* homolog *PlERS1* (Cluster-20879.39834), *OVA6* homolog *PlOVA6* (Cluster-20879.20949), *OVA7* homolog *PlOVA7* (Cluster-20879.36837), and *OVA8* homolog *PlOVA8* (Cluster-20879.38120) were up-regulated in normal ovules during Pl_5-Pl_7. Their expression level was significantly higher than that of aborted ovules, and was similar to the expression pattern of these genes in *Arabidopsis*, presumably with a similar regulatory pattern on ovule development of *P.ludlowii*.

### Key regulatory genes of ovule abortion regulate early ovule abortion in *Paeonia ludlowii*

Several *CesA* genes in dove trees are significantly up-regulated in aborted ovules compared to normal ovules, and the fibrillin content in aborted ovules is significantly higher than in normal ovules [67]. Similar to dove trees, the expression of the *CesA* homolog *PlCesA8* (Cluster-20879.67422) was significantly higher in aborted ovules than in normal ovules during the Pl_5-Pl_7 in *P.ludlowii*. This suggests that *PlCesA8* plays a vital role in ovule abortion in *P.ludlowii*, and that the gene could regulate cellulose synthesis, leading to early ovule formation and ovule abortion.

Many genes in the HD-Zip family are related to embryonic development [68], including *VvHDZ05*, *VvHDZ09*, *VvHDZ13*, *VvHDZ17*, *VvHDZ23*, *VvHDZ24*, *VvHDZ27*, and *VvHDZ28* in the HD-Zip I subfamily and *VvHDZ01* in the HD-Zip II subfamily. *VvHDZ01* in II was associated with grape embryo abortion [69]. In this chapter, the HD-zip transcription factor family homologs *PlHB14* (Cluster-20879.48787), *PlHB12L* (Cluster-20879.18846), and *PlHB7* (Cluster-20879.18847) were significantly highly expressed in aborted ovules of *P.ludlowii* and displayed low or no expression in normal seeds. This suggests that these three genes play an essential role in ovule abortion of *P.ludlowii*, and that their high expression in aborted ovules could affect the normal development of ovules and lead to ovule abortion.

*JMT* is a critical enzyme in the biosynthesis of MeJA, a jasmonic acid derivative. It is responsible for the methylation of JA into MeJA, which plays a vital role in plant seed development [70]. For example, *AtJMT* overexpression leads to increased MeJA accumulation and mediates stress signals leading to increased ABA levels, which affects the differentiation of rice spikes and ultimately leads to lower grain yields [71]. *AtJMT* overexpression leads to reduced seed numbers in *Arabidopsis* [72], while *PlJMT* (Cluster-20879.15575) was consistently up-regulated in aborted ovules of *P.ludlowii* and was highly expressed than in normal ovules. In *Perilla frutescens*, *PfJMT* gene expression was down-regulated as seeds developed [70]. The high expression of *PlJMT* in aborted ovules of *P.ludlowii* could affect ovule development and lead to abortion.

S-adenosylmethionine (SAM) is involved in various important physiological processes in plants, such as transaminopropyl, transmethylation, and transsulfuration, and is a critical methyl donor [56]. S-adenosylmethionine synthase genes (SAMs) are abundantly expressed in litchi aborted embryos, leading to embryo abortion in litchi [56]. SAM-related methyltransferase genes such as *LcCMT1*, *LcCMT2*, and *LcDRM2* have also been associated with 'Guiwei' litchi embryo abortion [73]. In *P.ludlowii*, SMT is a SAM-dependent methyltransferase, and the expression level of SMT2 homolog *PlSMT2* (Cluster-20879.38371) was sharply increased in aborted ovules, suggesting it plays an essential role in ovule abortion in *P.ludlowii*.

*EIL1* is a positive regulator of the ETH signaling pathway and primarily regulates the expression of most downstream ETH signaling genes [74]. *PbEIL1* is significantly up-regulated in seedless pears when seeds start to septate, and the ETH signaling pathway is activated, leading to excessive accumulation of ETH in the seeds and premature seed senescence and death. Its high expression in pears was associated with seed senescence in a transgenic experiment in tomatoes [75]. *PlEIL1* (Cluster-20879.37106) was consistently up-regulated in aborted ovules of *P.ludlowii* at the Pl_5-Pl_7 stage, and its expression was significantly higher than that of normal ovules. The expression pattern was similar to that of *PbEIL1*, indicating that *PlEIL1* plays a similar regulatory role in *P.ludlowii* as *PbEIL1* in seedless pears. The high expression of *PlEIL1* in aborted ovules may led to ovule abortion. All of these genes could be related to the defeat of *P.ludlowii*. However, the aborted functions of these genes must be further investigated. These findings will provide genetic resources for improving ovule abortion and expanding *P.ludlowii* production.

## Conclusion

In this paper, we investigated the characteristics and internal regulatory mechanisms of ovule abortion in *P.ludlowii* through physiological observations and transcriptome sequencing analysis. Transcriptome sequencing was performed on ovules at key stages during the early abortion in *P.ludlowii*, and the expression changes of key genes at different stages of ovule development were compared, including carbohydrate metabolism, phytohormone signal transduction, PCD, ovule development, and ovule abortion. Additionally, 15 key genes were screened for future research. We found that certain nutrients and phytohormones are essential for regulating ovule abortion, and that the exogenous application of nutrients and hormones could be an effective method of alleviating ovule abortion in *P.ludlowii*. This paper presents the first analysis of the mechanism of ovule abortion in *P.ludlowii*, which has significant implications for the conservation, propagation, and introduction of this species.

## Supplementary Information

**Additional file 1.** qRT-PCR primers information.

**Additional file 2.** Summary of Sequencing Data Quality.

**Additional file 3.**  GO enrichment analysis of DEGs.

**Additional file 4.** KEGG enrichment analysis of DEGs.

**Additional file 5.** DEGs involved in carbohydrate metabolism.

**Additional file 6.** DEGs involved in phytohormones signaling of *Paeonia ludlowii*.

**Additional file 7.** DEGs involved in PCD of *Paeonia ludlowii*.

**Additional file 8.** DEGs involved in the development ovule of *Arabidopsis* in *Paeonia ludlowii*.

**Additional file 9.** DEGs involved in ovule abortion of *Paeonia ludlowii*.

**Additional file 10.** DEGs involved in aborted ovules of *Paeonia ludlowii*.

**Additional file 11. Fig. 1.** Principal component analysis of this experimental samples.

**Additional file 12. Fig. 2.** Results of BUSCO evaluation of spliced transcripts (different colours represent different types of spliced transcripts).

## Data Availability

The datasets analyzed during the current study are available in the NCBI repository, the accession number to datasets: PRJNA818047.
