# Supplementary figures and images for: Transcriptome sequencing and gene expression analysis revealed early ovule abortion of Paeonia ludlowii
Source: BMC Genomics. 2023 Feb 17;24:78. doi: 10.1186/s12864-023-09171-1 (PMC9936667; doi:10.1186/s12864-023-09171-1)

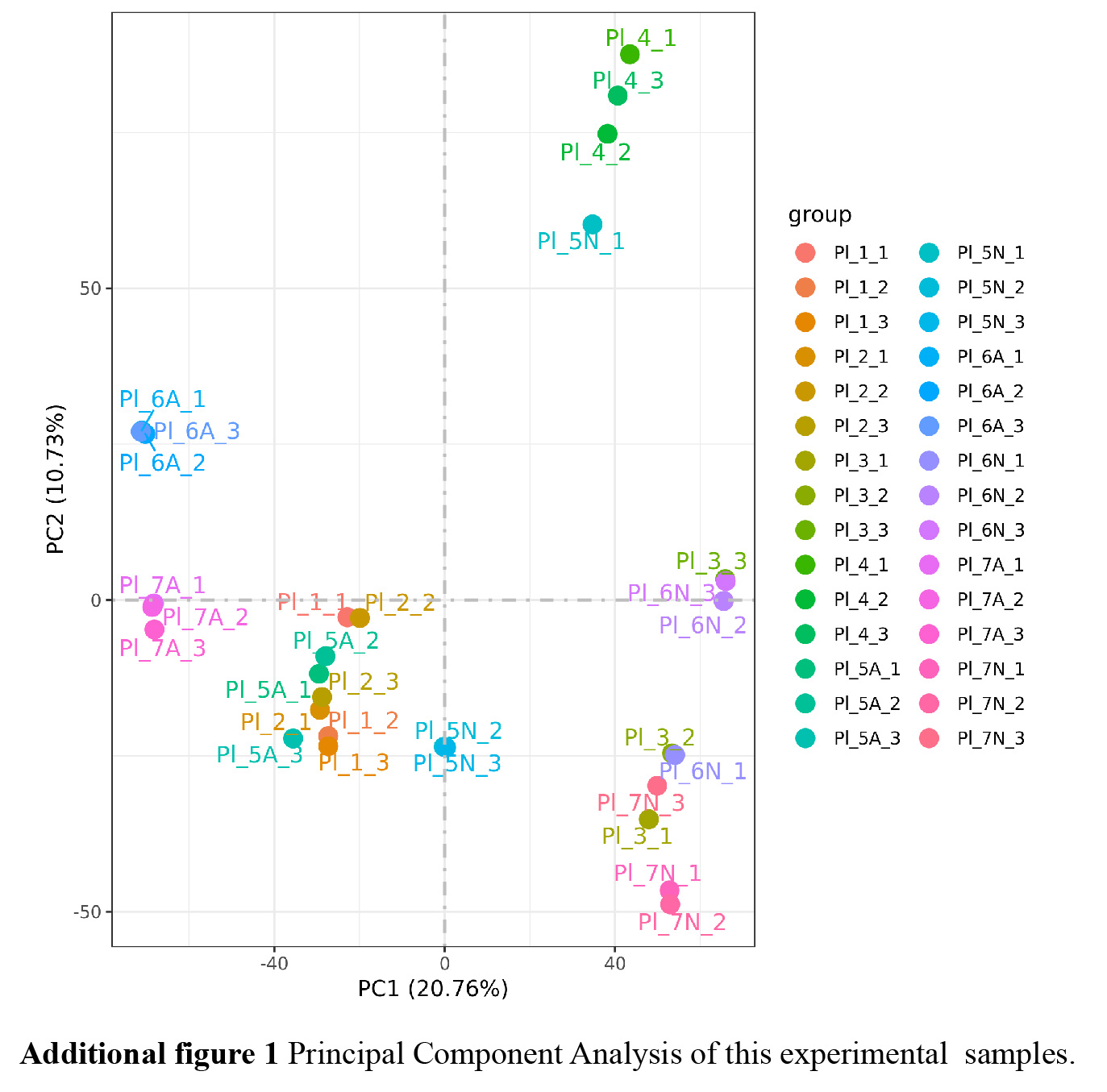

Supplement: Supplementary file 11 — Additional file 11. Fig. 1. Principal component analysis of this experimental samples. [file 12864_2023_9171_MOESM11_ESM.jpg]

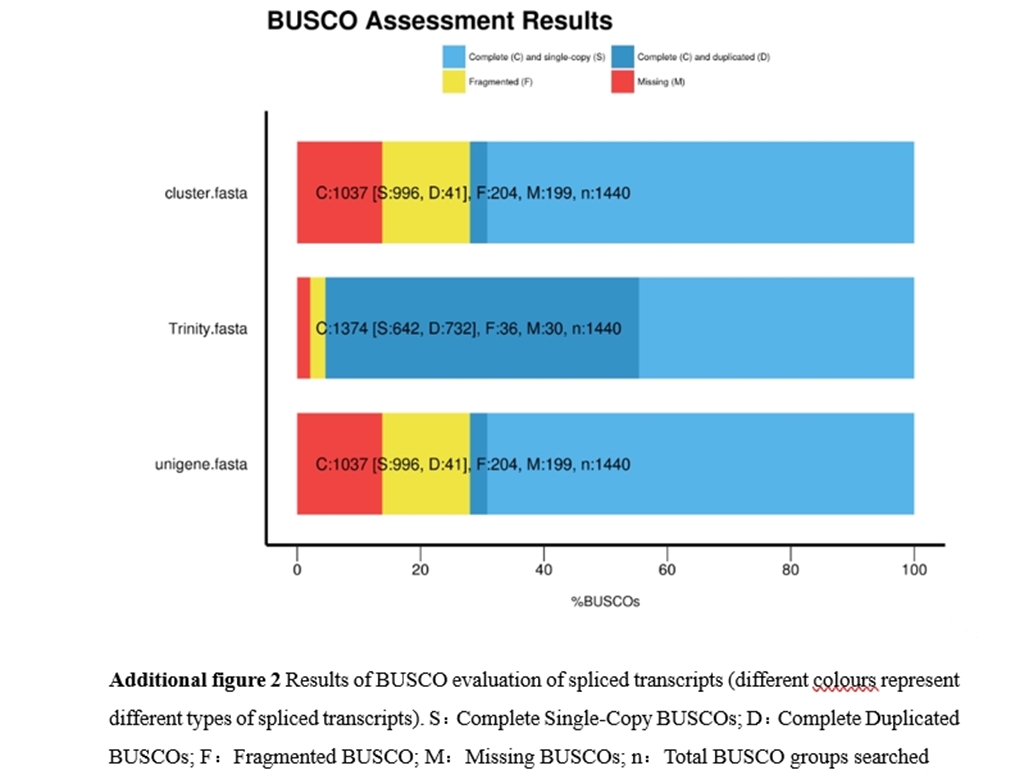

Supplement: Supplementary file 12 — Additional file 12. Fig. 2. Results of BUSCO evaluation of spliced transcripts (different colours represent different types of spliced transcripts). [file 12864_2023_9171_MOESM12_ESM.jpg]
